# Orthopædic Surgery

**Published:** 1896-02-15

**Authors:** 


### ORTHOPÆDIC SURGERY.

**Pott's Disease.**—For applying plaster of Paris jackets, Taylor[1] makes use of the following arrangement: The patient sits upon a bicycle saddle, and the feet rest in stirrups, which can be raised or lowered by a sliding joint and pin. The joint works on a rigid rod, which can be moved backwards and forwards, so that the patient's legs can rest in any desired position. An upright extends some distance behind the patient's head, and has a cross-bar at the top. The patient grasps adjustable handles, which are fixed to the vertical stem. The central upright is so attached to the saddle that it can be moved forwards in an antero-posterior direction, and thus be accommodated to the spinal curve. The object of the apparatus is to place the patient in as comfortable a position as possible while the jacket is being applied, and to secure a position which will elevate the ribs and produce a certain amount of lordosis involving both the dorsal and lumbar regions. In order that the jacket may fit over the pelvis, it is well to have the legs fully extended in the stirrups.

**The Horizontal Position for Applying Plaster Cast.**—T. W. Sloan.[2] The apparatus consists of a frame bearing a hammock. To the top rail of the frame a suspensory band is fixed, which can be raised or lowered at will by the operator. Around the patient and the hammock in which he lies, the plaster of Paris bandages are applied. In order to secure some extension of the spine, bandages are passed from the suspensory band above beneath the jacket, so as to in some degree support the patient and relax the tension on the hammock.

**The Treatment of Abscess.**—Tubby[3] after detailing the more serious aspects of abscess in Pott's disease, says that the methods of treatment open to us are five, namely: (1) The expectant, leaving the abscess to become encysted or absorbed; (2) aspiration; (3) aspiration, with the injection of fluids; (4) incision and drainage, and washing out the cavity with antiseptics; (5) the method advocated by Treves, Barker, and others; (6) dissection out of the sac. The conclusions arrived at are the following: Receding abscesses are best treated on the expectant plan; aspiration is of little use, and psoas and lumbar abscesses may often be cured by opening the sac, evacuating the pus, scraping the wall thoroughly, carefully drying the interior, and sewing it up; whenever possible, the lumbar should be preferred to the iliac or inguinal incision.

**Scoliosis.**—Forcible Massage as a Means of Straightening the Spinal Column: Dr. X. Delore (Lyons)[4] claims that he has employed this method with success in children. An anæsthetic is given, and if, for instance, the convexity be to the right in the dorsal region, the patient is placed on the left side. Cushions are put beneath the shoulder and pelvis, and considerable pressure is exerted on the right costal convexity by sudden and interrupted movements, the operator endeavouring to break the adhesions, mobilise the joints, and straighten the curvature of the vertebral column. Dr. Delore says that in children, and in his hands, no untoward accidents have occurred, but adds it is self-evident this variety of massage must be carefully and prudently applied. Sometimes the curvature is immediately and markedly straightened, while at other times no result at all appears to have been obtained. The Cause of Rotation in Scoliosis: Keetley[5] says if it be granted that the prime cause of scoliosis is, in young children, ordinary rickets, and in older ones rhachitis of adolescence, the situation of the epiphysial cartilages, which are the seat of the most extensive changes in these diseases, becomes important. Those above and below each vertebral body affect the anterior part of the spinal column more than the posterior, and this is the prime cause of tortion, that is to say, excessive growth at the anterior part of the bodies rather than the posterior makes them deviate laterally and so rotation arises.

**Pathological Anatomy of Scoliosis.**—Hoffa[6] gives in detail changes which he has found in scoliotic spines, but he says that the architecture of the spine in this deformity depends entirely upon the statical conditions. The obliquity of the position of the intraosseous columns shows the direction of the force which is acting upon the bone.

**Congenital Dislocation of the Shoulder.**—Phelps[7] succeeded in reducing the head of the humerus by removing a portion of it and clearing out the glenoid cavity. The patient was a boy nine years old, who, after birth, the labour having been instrumental, was found to have dislocation of the humerus backward beneath the spine of the scapula.

**Congenital Absence of the Radii.**—S. L. McCurdy.[8] The patient was aged five months. McCurdy discarded Bardenheuer's method of replacing the defect at the lower part with bone, so he split the ulna transversely, and attached the upper part of it to the semilunar bone, with a good result.

**Congenital Dislocation of the Hip.**—E. H. Bradford[9] concludes that the methods of treatment by traction or by mechanical means, with or without tenotomy, do not effect a cure; that correction by forcible reduction without incision is inapplicable in but few cases, and is not reliable; that the method of operative reduction offers the best prospect of a cure, though it is not at present without risk. T. Halsted Myers[10] thinks that Paci's method by manipulation is to be tried at any age. Failing this, injections of chloride of zinc and, if necessary, Hoffa's or Lorenz's operation, if the child is not over ten. Dr. Myers reports very satisfactory improvement by traction, immobilisation and protection, both in his own practice and in that of others.

**Incurvation of the Neck of the Femur in Adolescence.**—Royal Whitman[11] gives a very clear account of this affection, the symptoms being that the trochanter becomes elevated above Nélaton's line, the foot is everted, abduction and internal rotation are limited, and shortening of the limb is noticed. From congenital dislocation of the hip it is diagnosed by the absence of the up and down movement of the trochanter when traction is made on the foot. In severe cases osteotomy is necessary below the trochanter.

**Club-foot.**—R. L. Swan[12] having found that many cases do not become entirely cured by treating the foot alone, on account of the inward rotation which exists in the entire limb, performs an osteotomy on the tibia and fibula and brings the foot to the front. E. Muirhead Little[13] has done a new form of operation on the bones for resistant talipes equinus in adults and adolescents. It consists in the removal of a wedge from the neck of the astragalus and the os calcis, the wedge has its base upwards and its apex situated at the under part of the os calcis. For talipes valgus in the adult, James E. Moore[14] advocates in resistant cases the removal of the astragalus. He quotes one case, but the results do not seem to have been very satisfactory.

**Arthrodesis.**—Robert Jones,[15] of Liverpool, reported some months since fifteen cases, in which he has ankylosed the knee or ankle or both for severe infantile paralysis. Considerable improvement in the use of the limb was noted in nearly all the cases. The subject is further discussed by Dr. Ferdinand Karewski.[16]

**Flat-foot.**—Ricketts,[17] from a comparative and clinical study of flat-foot, comes to the following conclusions: That it is due to one of three causes—the failure of the tissues to become arched, rickets, and trauma. He states that the anthropoid apes have little or no indication of an arch, that but little benefit can be derived except in the lesser degrees of deformity, and that the aggravated forms of flat-foot should be subjected to the operation of Trendelenburg or of Gleich. Finally, he says, that his own experience would lead him to operate unhesitatingly upon any flat-foot causing incapacity by pain or deformity which could not be relieved by mechanical appliances.

**Dislocation of the Peroneus Longus Tendon.**—Mr. Walsham[28] gives the symptoms and causes of this affection, and then discusses treatment. He alludes to the uselessness of placing a crescent-shaped graduated pad over the tendon, and also decries division of the tendon on the ground that the function of the tendon is lost. He has made in one case a new sheath for the dislocated tendon by turning down the flap of periosteum from the lower end of the external malleolus, carrying it over the tendon, and suturing it to the fibrous tissue lying at the back of the normal groove.

**Morton's Metatarsalgia.**—A case is described by O. T. Osborne and W. H. Carmault.[19] After speaking of the causation and symptoms of this affection, the full account of their case is given. The usual methods fail to relieve it, but the patient was completely cured by excision of the head of the fourth metatarsal bone.

**Hammer-toe.**—Péraire[20] describes a means of curing this affection by cuneiform osteotomy of the head of the first and base of the second phalanx. He finds that local anæsthesia by means of injection of cocaine or ethyl chloride is sufficient. The indications for the operation are stated to be ankylosis of the articulation between the two phalanges, the presence of a corn or inflamed serous bursa, due to friction of the boot over the projecting part of the toe, and pronounced lameness.

**Ankylosis.**—Howard Marsh[21] combats the generally received opinion that suppuration in a joint is necessarily followed by bony ankylosis, and that there is only one cause for it, viz., suppuration. After acute purulent inflammation, if efficient treatment is employed directly the pus is suspected, in the majority of cases fibrous and not bony ankylosis follows. He states also that bony ankylosis often occurs in joints, independently of the suppurative process, and may be seen in the following groups, viz., in tuberculosis, especially in the spinal column, occasionally in Charcot's disease, in gout and gonorrheal rheumatism, in scoliosis and in osteitis deformans, also in osteo-arthritis. The article is very instructive, and well repays perusal.

[1] N. Y. Med. News, No. 1,158, p. 317. [2] N. Y. Med. Record, Aug. 10th, 1895. [3] The Hospital, Nov. 2nd, 1895, p. 79. [4] The Medical Week, Aug. 23rd, 1895. [5] Lancet, No. 3,751, p. 147. [6] Verhaud. d. Deutsch. Gesellsch. f. Chir. XXII., Congress, 1894. [7] N. Y. Med. Record, Sept. 21st, 1895, p. 427. [8] N. Y. Med. Jour., Sept. 19th, 1895. [9] Annals of Surg., Aug., 1894. [10] Ibid. [11] Trans Amer. Ortho. Assoc., 1894, Vol. VII. [12] Dublin Jour. Med. Science, Oct. 1st, 1895. [13] Brit. Med. Jour., Oct. 19th, 1895. [14] N. Y. Med. Jour., Sept., 1895. [15] Prov. Med. Jour., Dec., 1894. [16] Central f. Chir., Sept. 7th, 1895. [17] Therapeutic Gazette, Oct. 15, 1895. [18] Brit. Med. Jour., Nov. 2nd, 1895. [19] Yale Med. Jour., Vol II., 1895, No. 1. [20] Revue de Chirur., July, 1895. [21] "The Pathology and Clinical History of Some Rare Forms of Bony Ankylosis," Brit. Med. Jour., Nov. 2nd, 1895.

